# The Current Landscape of Artificial Intelligence in Positron Emission Tomography (PET) Imaging Across the Cancer Continuum

**DOI:** 10.3390/jcm15062446

**Published:** 2026-03-23

**Authors:** Wut Yee The Zar, Mi Rim Kim, Aruni Ghose, Sola Adeleke, Manoj Gupta, Partha S. Choudhary, Anirudh Shankar, Srishti Mohapatra, Stergios Boussios, Akash Maniam

**Affiliations:** 1 Department of Medical Oncology, Portsmouth Hospitals University NHS Trust, Portsmouth, UK; 2 Velindre Cancer Centre, Velindre University NHS Trust, Cardiff, UK; aruni.ghose1@gmail.com; 3 CanPrecise AI, Kolkata, India; anirudh2406@gmail.com; 4 Department of Research and Innovation, Medway NHS Foundation Trust, Gillingham, UK; 5 European Interdisciplinary Society for AI in Cancer Research, Milan, Italy; 6 Department of Cancer Imaging, School of Biomedical Engineering & Imaging Sciences, Faculty of Life Sciences and Medicine, King’s College London, London, UK; 7 Cancer Centre at Guy’s, Guy’s and St Thomas’ NHS Foundation Trust, London, UK; 8 Department of Nuclear Medicine, Rajiv Gandhi Cancer Institute and Research Centre, Delhi, India; manoj.gupta.dr@gmail.com (M.G.); pschoudhary@hotmail.com (P.S.C.);; 9 Diagnostics, Data and Digital Health Programme, School of Engineering, University of Warwick, Coventry, UK; 10 Artificial Intelligence and Machine Learning Programme, Department of Computer Science and Engineering, Ramaiah University of Applied Sciences, Bangalore, India; 11 Department of Medical Oncology, Ioannina University Hospital, Ioannina, Greece; 12 Faculty of Medicine, School of Health Sciences, University of Ioannina, Ioannina, Greece; 13 AELIA Organisation, 9th Km Thessaloniki—Thermi, 57001 Thessaloniki, Greece; 14 School of Cancer and Pharmaceutical Sciences, Faculty of Life Sciences and Medicine, King’s College London, London, UK; 15 Faculty of Medicine, Health and Social Care, Canterbury Christ Church University, Canterbury, UK

**Keywords:** artificial intelligence, PET, oncology, diagnosis, theranostics, federated learning, machine learning, physics-informed AI, hybrid models, image reconstruction

## Abstract

PET scans have long been used in oncology imaging to provide molecular and metabolic information about diseases. The use of artificial intelligence (AI) in PET scans in oncology theranostics has the potential to optimise PET modality and overcome the constraints that PET scans have, such as semi-quantitative metrics, reader subjectivity, and variability across scanners/institutions. Advances in AI and radiomics are overcoming those limitations by deep learning lesion detection, enhancing image reconstruction, and improving noise resolution, which allows ultra-low dose acquisitions, while physics-informed models integrate with PET systems to strengthen interpretability and quantitative accuracy. There are also predictive AI frameworks that link PET imaging biomarkers to therapy response and outcomes, create individualised care and are even able to simulate treatment response and help with treatment planning. However, challenges do exist. Most AI PET studies are retrospective, single-centre, and underpowered (small sample), with limited external validation and inconsistent standardisation (in acquisition, segmentation, and extraction), leading to poor reproducibility and higher performance estimates. Furthermore, ethical considerations, including data protection and transparency, need to be considered before implementation. Federated learning, physics-informed frameworks, and adherence to standardised protocols offer steps towards regulated AI systems. In summary, PET is evolving from an imaging modality to a platform with the integration of deep learning, radiomics and reconstruction capable of predicting treatment response and guiding treatment. With rigorous prospective validation, cross-institutional collaboration, and regulatory standardisation, AI in PET would create an advancement in nuclear medicine imaging in oncology.

## 1. Introduction

In oncology, Positron Emission Tomography (PET) has developed into a crucial molecular imaging platform for detecting the metabolic and receptor-level processes that aid in the diagnosis, staging, and treatment of cancers. Over the past three decades, continuous innovation—from PET/CT to time-of-flight and total-body PET—has enhanced image quality and quantitative accuracy [1]. These improvements have further enhanced the modality’s applications from static lesion detection to dynamic, system-level assessment of tumour biology. However, despite its improvement, PET is still limited by semi-quantitative methods of interpretation, scanner variability, and limited reproducibility. Recently, the application and advancement of Artificial Intelligence (AI) in the PET imaging workflow could potentially reduce those limitations. AI techniques such as machine learning, deep learning and radiomics can automate the identification of lesions, enable reconstruction from low-dose images, standardise delineation and segmentation, and identify predictive biomarkers that accrue imaging and molecular profiles. Therefore, a shift from PET being a qualitative diagnostic procedure towards a quantitative and predictive modality at the centre of personalised management of cancer disease is highly possible in the future.

More generally, Artificial Intelligence (AI) has been gradually incorporated into current medicine, especially in medical imaging; in this area, algorithms that use data are applied to support image interpretation, automate workflows and aid in clinical decision making. Machine Learning (ML) enables systems to find patterns in the data they are trained on. Deep Learning (DL) is a type of machine learning that uses multi-layered neural networks to model complex, non-linear relationships between variables. Both machine learning and deep learning have been applied extensively throughout all areas of oncology for both diagnostic purposes and treatment planning/prediction of outcomes and have created challenges with regard to interpretability, bias, validation and governance.

In comparison to the various reviews of individual AI applications (e.g., radiomics or reconstruction), this review will provide an end-to-end, integrated perspective on AI in PET imaging for cancer patients from reconstruction through to diagnosis, prediction, theranostics and regulatory approval as well as validation maturity, barriers to translation and some new approaches to the field including physics-informed AI, federated learning, and digital twins, which have been less emphasised in prior PET-specific reviews.

This review was carried out in the form of a narrative literature review. The studies were found through focused searching of PubMed, Web of Science, and Google Scholar with combinations of search terms that included PET, artificial intelligence, radiomics, deep learning, reconstruction, theranostics, and oncology. Preference was given to original research reported in the peer-reviewed literature; when possible, preference was also given to multicentre studies and to recent high-impact reviews. Excluded from this review were conference abstracts, PET applications outside of oncology, and technical-only studies with no clinical application. A formal systematic methodology such as PRISMA was not used, but attempts were made to minimise the selection bias of this review by including studies representative of all aspects of PET, including diagnostic, reconstructive, predictive, and regulatory.

## 2. Key Features of Radiomics and Deep Learning in PET Imaging

Radiomics is a process in which features are manually defined and extracted from PET images to quantify tumour characteristics such as texture, shape and intensity [2,3]. These manually crafted features require a high degree of standardisation in the acquisition, segmentation and preprocessing of the images. Differences in imaging parameters or software between different centres lead to differing process results. Therefore, the radiomics process is dependent on methodological homogeneity, meaning that consistent imaging protocols, segmentation methods and feature-extraction pipelines are required to ensure that results are comparable and reproducible across institutions. These concerns were further reinforced by the fact that, even though PET radiomics has the potential for use as a tool for personalised medicine, it is limited due to variability with respect to acquisition, segmentation, and reconstruction of the imaging data used to develop the features, which impact both feature stability and reproducibility. There is also a significant need for the development of standardised radiomic workflows and for multicentre validation to facilitate clinical application of PET radiomics [4].

A major limitation of PET radiomics from a nuclear medicine perspective is feature instability. Features produced by radiomics can fluctuate dramatically due to a variety of factors, including reconstruction algorithms (e.g., OSEM, Ordered Subset Expectation Maximisation vs. BSREM, Block Sequential Regularised Expectation Maximisation), voxel resampling, smoothing filters, discretisation methods, and tumour segmentation. Test–retest and reconstruction studies show that some of these texture features can dramatically shift based on the technical specifications used at the time of image production, even though tumour characteristics remain unchanged [4]. If a radiomic feature is more sensitive to acquisition or reconstruction parameters than to true tumour heterogeneity, its biological interpretability and clinical relevance are questionable. Although the use of IBSI (Image Biomarker Standardisation Initiative) standards for feature definition improves the consistency of the definition of those features [2], IBSI standards do not provide an assurance that radiomic feature definitions will be consistent across scanners or imaging protocols. Accordingly, it is necessary to conduct rigorous test–retest validations and demonstrate biological correlations prior to PET radiomic signatures being interpreted as clinically relevant.

The methodology of distinguishing between the three different radiomics paradigms—handcrafted, deep learning and end-to-end is significant. Handcrafted radiomics involves extracting pre-defined, quantifiable features (for example, first-order statistical parameters derived from image intensities, matrices of texture features, and descriptors of tumour volume) from manually segmented tumour volumes. The generation of these features is based upon mathematical definitions that require standardised preprocessing. Deep learning-derived features represent a type of latent representation that is learned through the application of convolutional neural networks. Handcrafted radiomics require tumour segmentation and can be applied to smaller datasets, while deep learning models require larger datasets but can analyse whole images without segmentation. Handcrafted features are also generally more interpretable because they correspond to image characteristics such as intensity, texture and shape. These representations can then be combined with either handcrafted radiomic or clinical variables in so-called hybrid or “deep radiomics” models. End-to-end deep learning models involve no explicit feature engineering as they map input (raw or minimally processed PET images) directly to an output (a clinical determination). As such, the above distinctions have significant implications in terms of how features are interpreted, what the required characteristics of data are, how robust models can be developed, and what validation strategies should be employed [2,5,6].

In contrast to handcrafted radiomics, deep learning utilises neural networks that automatically learn relevant patterns from the raw image data without the need for manually engineered features [5]. This provides the possibility of end-to-end prediction and reconstruction of images with minimal human intervention. The reduced dependence on human intervention minimises operator bias and allows the model to work directly from the data. However, such models require substantially larger and more heterogeneous datasets for obtaining reliable performance and generalisability.

Currently, the majority of the literature regarding the use of radiomics and machine/deep learning algorithms is limited to methodological and “proof-of-concept” studies; therefore, there is a great deal of need for rigorous validation, robustness testing and biological correlation prior to using radiomics-derived models as a reliable clinical decision-making tool in paediatric cancers. Large collaborative initiatives are beginning to address these challenges. For example, the European PRIMAGE (PRedictive In silico Multiscale Analytics to support cancer personalised diaGnosis and prognosis, Empowered by imaging biomarkers) project has developed a multi-institutional AI platform integrating imaging biomarkers, clinical data, and molecular information to support diagnosis and prognosis in paediatric cancers such as neuroblastoma and diffuse intrinsic pontine glioma [7].

Recently, the advancements in mixed or “deep radiomics” methodologies have combined deep learning-derived features with conventional radiomic variables. These hybrid models provide the interpretability of hand-engineered radiomics with the representational capacity of deep neural networks, enhancing predictive performance. Furthermore, hybrid PET/CT systems can predict immune microenvironment characteristics, such as CD8^+^ T-cell infiltration [8]. They can predict long-term outcomes in breast cancer using hybrid deep, radiomic and clinicopathological features [9]. Similarly, hybrid techniques have improved the prediction of treatment response in lung cancer using deep-radiomic mixing techniques [10]. In summary, deep radiomics is a hybrid framework that improves several characteristics in model robustness, interpretability and translational value in oncological PET imaging. Upon reviewing these AI models, radiomics and deep learning can be regarded as complementary paradigms in PET analysis based on artificial intelligence (AI). The radiomic approach involves the quantitative extraction of predefined features linking imaging phenotypes to their underlying molecular and biological characteristics. It allows the objective measurement of tumour heterogeneity and behaviour. In contrast, deep learning comprises the data and representations derived directly from raw images in optimising feature learning, aiming for automation, image reconstruction and improvement of diagnostic quality. If these two areas are combined (e.g., within hybrid or “deep-radiomic” frameworks), their combined strengths could facilitate some changes in PET, which could convert this largely qualitative imaging modality into an entirely predictive and individualised tool for oncological assessment and treatment planning.

## 3. Diagnostic Limitations of PET and AI for Lesion Detection and Classification

Positron Emission Tomography has been one of the main imaging techniques in oncology. However, its accuracy is still affected by several issues, such as subjective reading of images, use of semi-quantitative parameters involving SUVmax, and inter-scanner variability. These factors can cause issues with lesion detectability, reproducibility and distinguishing between different pathologies with similar metabolic behaviours.

Recent work in the artificial intelligence literature has begun to provide some solutions by automating the analysis of images, evaluating radiomic features, and improving classification accuracy. For example, advances in deep learning-based lesion detection have demonstrated that convolutional and multimodal networks can be used to fuse PET and CT data for more accurate tumour delineation and response prediction [3]. Another example is the measures using several machine learning techniques to evaluate whether they could detect mediastinal lymph node metastases from non-small-cell lung cancer (NSCLC). It was reported that convolutional neural networks (CNNs) were equal in accuracy to human expert radiologists and outperformed traditional feature-based models [11].

However, the study was limited in generalizability because it was retrospective and performed in a single centre. It included a small total number of nodes (1397) and no real external validation, raising the question of the reliability of the models.

Likewise, breast carcinoma and lymphoma differentiation using 18F-FDG PET/CT radiomics shows high accuracy, with an area under the curve (AUC ≈ 0.87–0.89) [12]. However, it had a small sample size, and a biological correlation was absent. Therefore, it is regarded as a technical proof-of-concept and not yet potentially clinically applicable. This work was extended by integrating data preprocessing and ensemble learning to classify breast tumour subtypes [13]. Their methodological rigour stems from the fact that product-level standardisation enables PET radiomics to enhance reproducibility. Still, the investigation emphasised that even finely tuned machine learning models have difficulty achieving high precision in subtype receptor-level classifications. This highlights the biological intricacy that cannot be overcome with PET imaging alone. Therefore, even advanced AI-driven radiomic models cannot fully reproduce receptor-level classifications, indicating that PET imaging alone cannot substitute molecular profiling.

In another interesting study, researchers focused on the immune-connected tumour microenvironment (the tumour microenvironment includes immune cells (like CD8^+^ T-cells), blood vessels, fibroblasts, and signalling molecules—all of which influence tumour growth and response to therapy). They developed a new diagnostic morbidity ground by associating radiomic features with the tumour micro-environment of immune cells, developing a hybrid PET CT clinician model. Their model could predict CD8^+^ T-cell infiltration with an AUC of 0.93, meaning it predicted CD8^+^ T-cell infiltration with very high accuracy [8]. This is a step towards biologically interpretable AI in imaging. However, the authors noted that their results depend heavily on their institution’s imaging protocols—scanner type and reconstruction parameters.

The role of AI was further explored regarding lesion detection and segmentation. A review of the application of AI in PSMA PET/CT imaging was done for prostate cancer [14]. It indicated that the performance of AI-based lesion detection models is like human experts, which suggests that AI can help standardise interpretation. The main barriers are a lack of external validation and regulatory barriers. To address the issue of data sharing and generalizability, they proposed a federated learning framework. Federated learning allows multiple centres to train a shared AI model without exchanging raw patient data to maintain confidentiality. Each site trains a model locally, with only the model weights (learned parameters) being shared, not the scans themselves. However, it demands high computational infrastructure and careful coordination between centres, which in the future will be among the ongoing challenges of AI use in PET. This model’s validation was also limited to specific cancer types, and it has not yet proven generalisation across all cancers.

A more abstract, translational outlook showed that AI-based PET measures can all accelerate drug discovery, immunotherapy, and biomarker development [15]. They were exploring how AI-enabled PET could bridge basic research and clinical application. However, like the studies, it was a scoping review of early studies, rather than reporting validated clinical translational outcomes.

In summary, the investigations collectively provide evidence that AI is transitioning the use of PET from a morphologic imaging modality towards the use of a quantitative and biologically applicable imaging modality (showing tumour metabolism, immune context, or treatment response). There are clear improvements in diagnostic accuracy, tumour characterisation, and reader consistency.

## 4. Reconstruction and Efficiency

The next topic included in this literature review is how AI is revolutionising PET image reconstruction and image quality. Traditionally established PET image reconstruction techniques are OSEM (Ordered subset expectation Maximisation) and BSREM (Block Sequential Regularised Expectation Maximisation). They have struggled with a trade-off between noise, spatial resolution, and complexity, which limits efficiency and reproducibility in the clinical setting. AI has developed into a solution for reconstruction in terms of speed and accuracy. AI-driven reconstruction, using deep learning architectures, overcomes these barriers by producing high-quality images from Ultra-low-dose or short-duration acquisitions. For example, the CycleGAN (generative adversarial networks) was able to transform or synthesise full-dose whole-body PET images from images with scans at an ultra-low dose (only one-eighth of the dose limit) [16]. Their model resulted in similar lesion detectability and had an SUV bias of <0.1. This is impressive because SUV accuracy is essential for quantitative PET—it affects staging, response treatment, and prognostic evaluation. In this study, two AI models used for reconstruction in PET were compared: the CycleGAN (CGAN) and ResNet (RNET). Of the two, CGAN produced higher quality than RNET, which showed that it preserved image detail and reduced noise, producing images like those of full-dose PET. The strengths of the study were the dataset and external validation. Both are rare strengths in AI imaging research. The limitation is that they used a single scanner and a single radiotracer, like FDG, which limits generalizability. It emphasises the need for multicentre, multi-tracer validation for broader application. Other limitations include possible observer bias, as the physicians were able to print out the LD images.

In traditional PET workflows, image quality control depends on human experts checking noise, artefacts, and resolution—a subjective and time-consuming process. To combat this issue, an AI-based tool was developed to automatically assess PET image quality, ensuring that reconstructed scans meet diagnostic standards [17]. They used a DenseNet-based architecture, which is a type of CNN. An automated AI-based evaluator ensures consistent quality and detects poor reconstruction early. Their result had an AUC of 0.92, meaning it could accurately distinguish between high-quality and suboptimal images with strong performance. Their study involved multiple institutions, showing that the method could work across different clinical settings—an encouraging sign of generalizability.

However, despite the multi-institutional setup, the data size was very small, which limits confidence in findings. With a small dataset, a deep learning model risks bias and model overfitting, as it does not have enough diverse examples to learn the full range of variability that exists in real-world PET images (e.g., scanner types, patient anatomy, noise patterns). As a result, the model tends to memorise the patterns and noise present in the training data instead of learning the underlying generalizable features that distinguish good- and poor-quality scans.

In this regard, deep learning PET imaging-enhancement algorithms (models designed to improve image quality and detail after reconstruction) that relied on radiomic features were investigated [16]. They compared the AI-enhanced PET images against the gold standard BSREM. The results showed that over 80% of the quantitative features were concordant with the BSREM baseline. This implies that deep learning methods can preserve quantitative quality while being more efficient. It is an essential point for clinical PET where radiomics consistency affects diagnosis, treatment response assessment and radiotherapy planning. The limitation is that the deep models may have “over-enhanced” certain features. This finding underscores the need for careful validation of AI-enhanced PET for diagnosis/clinical reliability.

To further cite their work, AI for PET reconstruction should integrate physical models of image formation, such as the physics of photon detection, attenuation, and noise, rather than replacing them entirely with deep learning. This approach is called “physics-informed AI” [5]. Complexities in PET image reconstruction include restricted spatial and temporal resolution, image noise and the aim to shorten scanning duration to decrease radiation exposure. The current reconstruction techniques depend on mathematical and physical models but fail to utilise the complete potential of available data. Deep learning AI enables the analysis of PET image manifolds to understand valid PET image characteristics and use them to enhance clarity and denoise images and for better data utilisation. The internal operation of AI systems remains unclear to users because they are often mistakenly viewed as “black box”-like systems. The clinical world faces issues of scepticism about its accuracy and reliability because of its complexity. The medical field is trying to overcome this by implementing AI to established physics-based models to enhance both decision-making in a more transparent manner and to assess reliability for further clinical applications. PET reconstruction benefits from three distinct applications of AI. (1) Direct AI method, which maps raw data but is limited by the need for enormous data and computational demands. (2) Physics-informed AI, which uses deep learning in already existing reconstruction algorithms to improve image quality and reduces the “black box” problem by grounding AI decisions into pre-existing imaging physics. (3) Post-processing AI that enhances already reconstructed images by denoising, which is already being used like in SubtlePET.

**Figure 1:**
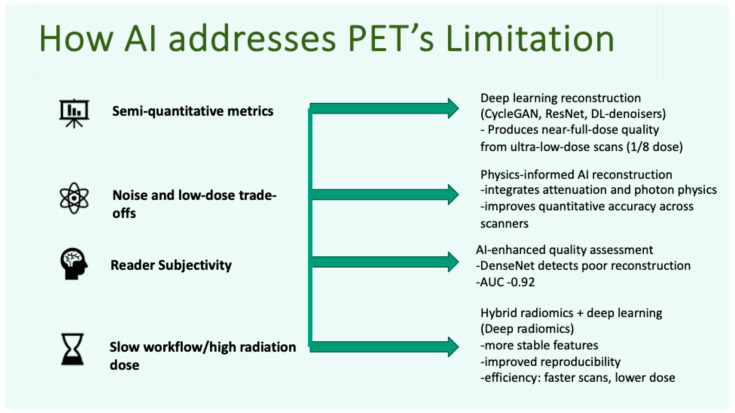
Overview of how AI addresses key limitations in PET imaging across the workflow, including image reconstruction (noise–resolution trade-off, ultra-low-dose acquisition), quantification (SUV stability and harmonisation), lesion detection and segmentation, and predictive modelling for treatment response and theranostics.

Overall, AI helps improve PET imaging quality; however, ensuring interpretability and clinical validation remain essential prior to widespread adoption (Figure 1).

## 5. Prediction and Personalisation

AI applications in PET/CT are moving towards prediction and personalisation in oncologic care. It is gradually extending beyond the primary imaging function to diagnose and stage disease and evaluate treatment response. Deep learning-trained radiomics have developed an ability to derive complex imaging biomarkers associated with molecular, immune phenotype, and prognosis. This transition represents a fundamental evolution in oncological imaging.

A deep learning model using 18F-FDG PET/CT that accurately predicted EGFR mutation status was developed [18]. They trained a deep convolutional neural network on PET/CT to learn visual and metabolic patterns associated with EGFR-mutant vs. wild-type tumours. The model achieved AUC 0.83–0.86 and successfully predicted progression-free survival in NSCLC patients. This suggested a potential for using imaging to support the selection of targeted therapy and immunotherapy, which moves PET into an era of precision oncology.

Similarly, we have previously discussed that a radiomics–clinical model was created based on PET/CT to non-invasively characterise tumour immune microenvironments, and to discriminate CD8^+^-high vs. CD8^+^-low phenotypes (AUC 0.93), supporting personalization with immunotherapy [8].

Deep learning was applied to PET/CT data for patients with clinically node-negative (N0) non-small-cell lung cancer (NSCLC) [19,20]. In many N0 cases, small or “occult” lymph node metastases may go undetected on routine imaging—leading to under-staging and suboptimal treatment planning. Their deep learning model analysed metabolic and spatial patterns on PET/CT to predict the presence of hidden (occult) nodal disease. They achieved AUC 0.88–0.96 in clinical stage N0 NSCLC; these results will likely guide surgical and adjuvant treatments.

Lastly, frameworks like the semi-supervised transfer learning approach were developed [21], and they achieved automation of tumour quantification and risk of progression modelling across multiple cancer types.

Both studies amplify the predictive and personalised potential of AI in PET imaging. Together they demonstrate AI’s capacity to enhance staging accuracy, automate tumour characterisation and inform individualised treatment strategies.

A synthetic PET from CT was developed to demonstrate the prediction of risk for malignancy and survival in lung cancer when PET is not available [22]. They use AI to generate synthetic PET images from CT data, creating “virtual PET” scans when PET imaging is not available. This is important because PET is expensive, limited in availability and requires radioactive tracers. Their findings are that the synthetic PET models were able to reliably estimate malignancy risk and survival. This underscores AI’s ability to integrate cross-modality information and blurs the line between structural imaging (CT) and functional imaging (PET).

All these studies demonstrate AI’s potential to reposition PET as a predictive, decision-support with non-invasive application to individualised oncology. However, most studies to date rely on retrospective, single-centre models with minimal biological interpretability and standardisation. They also again stressed the need for multicohort assessments to evaluate and integrate these predictive models in a clinical workflow, prior to being able to use them in clinical settings. As previously discussed, the predictive efficiency of hybrid PET/CT models has advanced in relation to predictions of treatment response and long-term outcomes. For non-small-cell lung carcinoma, a PET/CT-based model that combines radiomic features with clinical information showed high predictive power for pathological complete response to neoadjuvant treatment with a predictive accuracy AUC value of >0.90. This model provides a non-invasive method of pre-treatment stratification [10]. For breast cancer as well, a hybrid deep-radiomic model based on a combination of radiomic, deep learning and clinical parameters can effectively predict the five-year disease-free survival for those failing to achieve pathological complete response following neoadjuvant chemotherapy [9]. These studies showed that hybrid deep-radiomic models have the potential to make accurate predictions about treatment response and patient prognosis. This can lead to improving downstream personalised prognostic evaluation and treatment planning in a cancer care setting.

## 6. Theranostics and Future PET

AI applications in oncologic PET can be broadly categorised into (1) established clinical work enhanced by quantitative analytics, (2) early translational frameworks undergoing validation, and (3) forward-looking, hypothesis-generating concepts such as AI-enabled digital twins.

The frontier of PET theranostics is constantly shifting from static image interpretation to an AI-driven simulation of disease biology. Historically, PET scans have been utilised to visually interpret tracer uptake at a single time point. Clinicians would examine a set of static images and semi-quantitative values such as SUV (Standard Uptake Value) to measure the level of activity of the disease, response to therapy or progression of disease. With AI and enhanced computational models, PET imaging has advanced from merely snapshots to simulating biological processes, such as tumour metabolism, receptor expression or treatment response. An AI-enabled theranostic system can integrate imaging, clinical data, and multi-omics features to predict response and personalised treatment. This will mark a transition from diagnostic imaging to predictive and therapeutic planning support for clinicians [23].

Following the clinical validation of [^177^Lu]Lu-PSMA-617 VISION trial, which demonstrated survival benefits in metastatic castration-resistant prostate cancer, subsequent research now integrates deep learning with biological and physical models that characterise disease behaviour and radiotracer kinetics in the body. This means that AI can both learn imaging patterns and simulate the underlying tumour biology, helping to predict how each patient will respond to therapy. These developments mark a paradigm shift from static image interpretation to achieving patient-specific therapy optimisation. This is in alignment with the [1] roadmap that described a pathway toward “next generation” quantitative PET through next-gen detector physics, kinetic modelling, and AI-based reconstruction as part of the overall vision to achieve real-time, whole body dynamic imaging and true parametric quantification. The path laid out was predicated on the integration of precision dosimetry, systems biology, and AI, all three of which provide a foundation to develop digital twin-based theranostic approaches.

A roadmap for the use of digital twin systems in PET theranostics was proposed [24]. Data from PET scans, radiomic features and genomic and/or pathological data are integrated into a virtual twin of the tumour of each patient, which can learn and continually update itself. The deep learning models simulate the kinetics and radiotherapy delivery of the radiopharmaceuticals, whilst the generative models predict the distribution of tracers from the microscopic level to the whole body level. Thus, PET is likely to change from a static imaging modality to a dynamic interactive simulation environment for the planning and personalisation of treatment prior to it being delivered in the clinic.

The EANM (European Association of Nuclear Medicine) further explained regarding physics-informed foundation models (large-scale AI trained on diverse imaging and physical data to predict tracer behaviour and radiobiological response) and multi-agent AI architectures (interacting AI systems managing different tasks). Together, these systems would result in “computational nuclear oncology,” where PET data streams update virtual twins in real time to forecast response or toxicity. Despite their promise, these DTS (Digital Twin System) frameworks remain conceptual and are still limited by data heterogeneity, interpretability, and the need for rigorous prospective validation. Therefore, the concept of AI-based digital twins in nuclear oncology should currently be regarded as a forward-looking, conceptual framework rather than an established clinical tool. As we discussed earlier, evidence-based treatments like Lu177 PSMA 617 therapy in the VISION trial show that PET can directly guide cancer treatment. Additionally, advanced AI technologies discussed in [21] can simulate how a patient’s disease and treatment will behave. Uniting these two, the conclusion is PET in the future will be a smart system for predicting and personalising treatment. At present, these approaches are best viewed as hypotheses that require large-scale prospective validation and demonstration of outcome improvement before clinical integration.

## 7. Challenges and Validation Discussion

Despite good technical results for AI-PET from both a diagnostic and a reconstructive and predictive perspective, there are numerous methodological limitations that will need to be addressed before AI-PET can transition into the clinical setting. Most of the current literature evaluating AI-PET remains retrospective, single-site, and based on relatively small databases with little or no independent external validation. These types of study designs have an increased likelihood of overfitting and performance inflation (i.e., artificial increases in accuracy), limiting the generalizability of AI-PET findings across different scanner platforms, different institutions and different patient populations. Overcoming these limitations will likely require cooperative efforts among multiple sites and/or institutions working together to develop standardised imaging protocols and prospective, randomised clinical trial(s) to establish whether AI-PET significantly improves the diagnostic accuracy of PET scans.

Radiomics-based PET models have the highest degree of clinical readiness from a translational standpoint due to greater interpretability, less stringent data requirements, and some alignment with regulatory guidelines as they exist today. While end-to-end deep learning models are technically strong, their use is limited by their high data requirement and lack of transparency, which also contributes to variable levels of external validation. A hybrid approach (deep learning/radiomics) offers an intermediate position that can provide predictive capability with some level of interpretability; however, physics-informed AI frameworks are still primarily at the preclinical stage of development but have potential to improve both the robustness and regulatory acceptance of AI in PET imaging. Therefore, there are a few AI paradigms in PET imaging that are beyond the proof of concept and/or early translational stages of development and, therefore, not yet ready for routine clinical application.

Currently, most of the studies can be classified according to validation maturity. Studies approaching clinical readiness are scarce and require prospective validation and regulatory approvals. Proof-of-concept studies prioritise performance and feasibility, while translational studies extend further through multi-centre data and external validation but lack prospective evaluation. Hence, the use of PET AI is still at a primitive stage of translational development.

One of the main barriers to translating AI-PET to a clinical setting is the fact that PET imaging data are heterogeneous due to differences in both the hardware used for scanning and the processing methods (acquisition protocols) as well as image reconstruction algorithms and preprocessing methods utilised to process the raw data; these differences contribute to limited comparability and reproducibility of reported results by studies on AI-PET. It is important to note that negative and inconclusive results are under-represented in these studies; hence, a bias exists towards the positive results, overestimating clinical robustness and accentuating the need for independent validation. The AI PET model performance declines under external validation, especially across scanners, institutions or reconstruction protocols. These inconsistencies remain a barrier to generalizability.

Although prospective PET imaging trials are ongoing, few are specifically designed to evaluate AI algorithms as predefined clinical endpoints. This highlights a persistent translational gap between methodological innovation and clinical implementation.

One of the most extensive validation frameworks to date is the prototype for the FDA SaMD (Software as a Medical Device), through a multicentre deep learning model that predicted PD-L1 expression and immunotherapy response with their model based on PET/CT features [19]. It was the basis for scientific, analytical, and clinical validation, in retrospective and prospective cohorts, thus following full-cycle validation. While this is an exceptional example, it is again restricted to a single pathology, which raises the question of the reliability of the model for other conditions or pathologies. Table 1 summarises the representative AI-PET studies reviewed in this manuscript, including study design, validation strategy, and methodological limitations, focusing on sample size and external validation, as high accuracy in small retrospective or single-centre cohort studies does not translate into generalizability.

It is important to highlight that a lot of the studies summarised should be interpreted as proof-of-concept investigations. Studies with small sample sizes have a risk of overestimating performance and should not be interpreted as clinical readiness. They also limit generalizability.

Federated learning is a possible solution to the problems of data sharing and single-centre bias, by decentralising the training process to numerous different centres without the need for moving raw imaging data [25]. Federated learning frameworks and physics-informed reconstruction models require complex computational resources, including GPU infrastructure, secure network, and coordinated cross-institutional workflows, as well as data harmonisation, and integration with PET scanners. In this model, only the model weights, i.e., learned parameters, will be shared between centres while ensuring patient privacy is maintained, whilst achieving large-scale external collaboration. This decentralised model will optimise diversity of data and generalisability, resulting in better external validation across heterogeneous populations and imaging protocols.

Physics-informed AI (PI-AI) has been proposed to counter data heterogeneity and scanner-related variability by imposing established principles of the physics of PET imaging, e.g., photon detection, attenuation and noise models, within the learning process itself [5]. By anchoring deep learning architectures within physical models of image formation, PI-AI optimises fidelity of reconstruction performance and ensures quantitative parameters (SUV accuracy) can be preserved as reliable and comparable amongst systems. Together with this complementary innovation, it is possible to provide the scale and diversity for generalised model development by federated learning and provide the fidelity and standardisation required for quantitative clinical translation by physics-informed AI. This can potentially give a conceptual route towards reproducible, regulation-compliant AI implementation in PET imaging.

A systematic review of the reproducibility standards in AI research in radiology was conducted [26]. The finding is that fewer than 10% of studies provide open-source code, and only 6% of studies develop true external validation. Their review revealed how a lack of transparency, inconsistent declaration of hyperparameters, and focused single-centre data can lead to exaggeration of the purported model accuracy and transferability.

As it is previously discussed, AI studies are lacking standardisation and reproducibility. Structured reporting and benchmarking using standards such as the Radiomics Quality Score (RQS), Transparent Reporting of a multivariable prediction model for Individual Prognosis Or Diagnosis (TRIPOD), and Checklist for Artificial Intelligence in Medical Imaging (CLAIM) were advocated [2], with an emphasis that the quality of radiomics and deep learning studies should adhere to standardised protocols to ensure reproducibility and obtain regulatory approval.

The heterogeneity of data and cross-modal synthesis were reviewed [27]. One major problem is the cross-domain shift, when data from different scanners or hospitals does not look the same (different scanner hardware, reconstruction protocols, and preprocessing pipeline), and this is the reason AI models in PET fail to generalise. They were major sources of noise that can invalidate claims of reproducibility and bias validation, as data from different centres or imaging systems differ in their characteristics.

**Figure 2:**
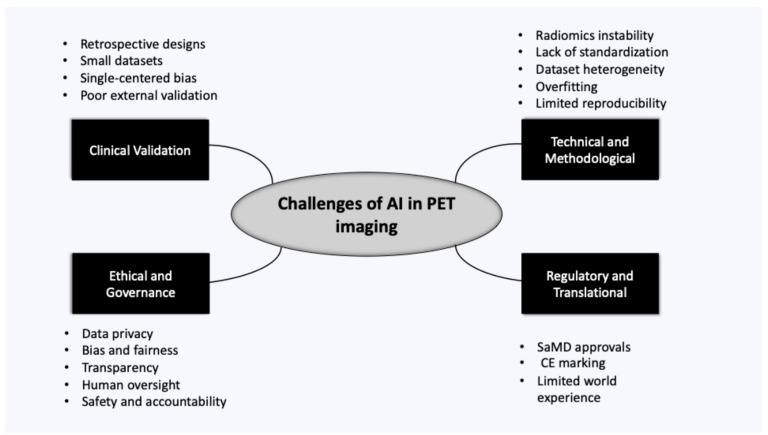
Mind Map of challenges of AI in PET imaging.

Overall, they suggested that even as technical innovation, such as federated learning and standards for reporting, narrow the gap in the reproducibility process, the issues of external validation and regulatory approval are not sufficiently addressed. The actual clinical expansion of AI-PET technologies requires open data and code practices, as well as standardised imaging protocols, to have consistent prospective multicentre validation using appropriate SaMD and CE marking. CE marking refers to the European degree of compliance with the Medical Device Regulation (MDR 2017/745), which indicates that AI-based medical imaging products are following safety, performance, and quality standards for their deployment in the EU (Figure 2) [28].

## 8. Ethical and Regulatory Considerations

Many of the specific regulatory concerns for PET are mirrored by those found throughout the broader oncology community concerning the use of Artificial Intelligence, such as transparency, post-approval surveillance, ongoing model updating, and cross-institutional validation. The evolving regulatory paths for the use of PET-based artificial intelligence in medical imaging are aligned with the emerging regulatory environment for all types of artificial intelligence software used in cancer treatment, including Software as a Medical Device (SaMD), adaptive algorithms, and monitoring of performance based on real-world data.

The regulatory framework governing the clinical application of AI in PET imaging is usually the regulatory framework of the SaMD (Software as a Medical Device). SaMD refers to the standalone software intended for medical purposes, including diagnosis, prognosis, or treatment decision, independent of dedicated hardware. Therefore, these AI-based PET systems are required to demonstrate analytical validity, clinical performance and ongoing risk assessment. CE marking (Conformité Européene) indicates conformity with European Union regulatory requirements under the Medical Device Regulation, certifying that a product meets essential safety and performance standards for clinical use. In Europe, CE marking under the MDR (Medical Device Regulation) 2017/745 requires that the medical device meet the minimum requirements regarding quality management, performance and safety before it can be deployed clinically. The recent review has emphasised that obtaining regulatory compliance by itself will not ensure reliable integration into clinical practice, especially since AI systems are rapidly evolving into multimodal and foundation model architectures. Transparency, governance of adaptive models and post-deployment performance evaluation have been increasingly recognised as key components of the responsible integration of clinical AI systems [29].

Beyond regulatory approval, ethical considerations remain the key factors in the use of AI in PET imaging. The most important of these are bias in the training data used for AI and a lack of transparency in how AI produces its results, as well as privacy concerns related to patients’ data and the need for accountable action to prevent harm to patients.

It has been indicated that AI tools could potentially provide unfair or variable results if the training data is biassed, i.e., if it does not contain a diverse representation of all populations or patient groups, making an uneven distribution of diagnostic or therapeutic options among patients a possible outcome [30]. Ensuring patient confidentiality and safe data sharing when developing large databases of medical images will also be necessary to protect patients from the risk of their personal information being compromised. A framework for ethically developed AI in healthcare has been proposed; that AI in healthcare should be fair, transparent, and patient-centred [31]. It has been emphasised that the responsibility for making decisions regarding patient care cannot be transferred from clinicians to AI systems, but rather, AI systems should provide explanations for their recommendations, and these should be continuously monitored by clinicians.

Ultimately, addressing the ethical and regulatory issues presented by AI-PET technologies, along with the technical validation of these technologies, will ensure the development of a responsible and reliable technology base for AI-PET in the field of oncology.

## 9. Conclusions

Artificial intelligence has rapidly changed positron emission tomography from a largely qualitative imaging technique to a quantitative, predictive, and possibly theranostic technique in the field of oncology. This review aims to critically examine how new and emerging approaches like physics-informed AI and digital twin systems integrate into the PET field, highlighting the gap that exists for clinical implementation.

In diagnostic, reconstructive, and predictive uses, AI has proven significant improvements in lesion detection, image quality, and individualised treatment planning. Hybrid radiomic–deep learning approaches, physics-informed reconstruction, and predictive deep models all point to the possibility for PET to enhance beyond static imaging, leading to dynamic and biologically interpretable tools. However, even in the face of these advances, substantial challenges and limitations are ongoing issues. Most studies examining the role of AI in PET are still retrospectively designed, single-centre, and poorly externally validated, resulting in a lack of standardisation in the acquisition approach and reporting structure. Heterogeneities across scanners, reconstruction pipelines, and datasets that adversely affect reproducibility still exist. The introduction of federated learning, the use of open data, and the use of standardised reporting guidelines such as RQS, TRIPOD, and CLAIM are critical to transparency, reproducibility, regulatory readiness, and risk mitigation.

Specifically, the lack of consensus regarding heterogeneity among imaging scanner vendors, reconstruction methods, and institutional protocol implementation continues to be one of the biggest impediments to both reproducibility and clinically deployable generalizability of AI-PET systems.

Overall, achieving clinical translation will depend on solid prospective multicentre validation, as well as regulatory alignment through frameworks such as that associated with SaMD and CE marking. The converging systems of digital twins, physics-informed AI, and large-scale collaborative networks are likely to improve PET as an intelligent imaging system that not only visualises disease but also simulates and optimises therapeutic responses. It is a promising future in which AI-directed PET imaging models will have a great potential to shape and change the future of precision oncology and computational nuclear medicine.

## Figures and Tables

**Table 1:** Overview of AI-based PET imaging studies in oncology, including study design, validation and methodological limitations.

Study (Primary Focus)	Reference	Sample Size/Dataset	Study Design	Validation Type	Key Methodological Limitation	Noteworthy Strength (Rigour)
DL image enhancement algorithms in PET/CT imaging: a phantom and sarcoma patient radiomic evaluation	Bonney et al., 2025 [18]	20 sarcoma PET scans	Retrospective single-centre study of DL vs. OSEM and BSREM	Phantom and clinical data validation (single-centre)	Very small sample size (*n* = 20): sarcoma-only cohort: limited clinical generalizability despite radiomic concordance	Combined phantom + patient validation; radiomics-based evaluation; >80% radiomic feature equivalence
DL model for automatic image quality assessment in PET	Zhang et al., 2023 [17]	89 PET scans (71 training, 18 validation)	Retrospective (supervised deep learning classification; single-centre)	5-fold internal cross-validation (single-centre)	Small dataset; no external validation; no brain scans; single scanner	High AUC (up to 0.91 in test set); reproducible; clinically applicable framework
DL-assisted ultra-fast/low-dose whole-body PET/CT imaging	Sanaat et al., 2021 [16]	100 patients	Prospective study (comparing DL models creating FD images)	External validation (single-centre)	Single-centre; FDG only; single scanner; limited assessment across tracers and reconstruction protocols	True low/full-dose data; external validation; 1/8th dose achieved clinical equivalence
A Machine Learning Model Based on PET/CT Radiomics and Clinical Characteristics Predicts Tumour Immune Profiles in NSCLC	Tong et al., 2022 [8]	TCGA: 1145 (RNA-seq); DPH: 221 PET; TCIA: 39 external	Retrospective multicohort study	Internal (Daping Hospital, DPH) + External (TCIA)	Retrospective; small sample size; incomplete TIME subtype coverage	High predictive performance (AUC = 0.93); external validation; links imaging to immune phenotypes
Radiomics based on 18F-FDG PET/CT could differentiate breast carcinoma from breast lymphoma	Ou et al., 2019 [12]	44 patients (65 lesions)	Single-centre retrospective study (differentiate between breast carcinoma vs. lymphoma)	Internal validation	Small sample; single institution; risk of overfitting preliminary results; proof-of-concept study; no external validation	PET/CT radiomics achieved AUC up to 0.89; integration of clinical + imaging data improved accuracy
Breast Tumour Characterization Using 18F-FDG PET/CT and Radiomics	Krajnc et al., 2021 [13]	170 patients (173 lesions)	Prospective single-centre study using supervised ensemble ML	100-fold Monte Carlo cross-validation (internal)	Single-centre; no external validation; moderate AUC for receptor status prediction	IBSI-standardized workflow; strong model reproducibility; high AUC for malignancy (0.81)
Multi-institutional PET/CT image segmentation using federated deep transformer learning	Shiri et al., 2023 [25]	328 from 6 centres (head and neck cancer)	Multi-institutional federated learning for segmentation	Multicentre federated learning (7 FL algorithms) vs. centralised	Single cancer type; computational complexity; no clinical outcome endpoint	True multicentre FL; matched centralized Dice (0.80); robust and generalizable
Comparison of ML methods for classifying mediastinal lymph node metastasis of NSCLC	Wang et al., 2017 [11]	168 patients; 1397 lymph nodes (127 malignant)	Retrospective per-node ML/DL comparison (RF, SVM, ANN, CNN)	10 × 10-fold cross-validation; compared with human	Single-centre; class imbalance; CNN not optimised for small nodes	Large dataset; direct ML vs. DL vs. radiologist comparison; CNN AUC = 0.91; showed automation potential
Deep Semi-supervised Transfer Learning for Fully Automated Whole-Body Tumour Quantification and Prognosis on PET/CT	Leung et al., 2024 [21]	1019 patients (611 FDG-PET/CT, 408 PSMA-PET/CT) across 6 cancer types	Retrospective, multi-cancer, multi-tracer	Internal + external validation	Retrospective; heterogeneous data sources; class imbalance; limited low PSMA-RADS cases	Large, multi-disease dataset; automated segmentation and prognosis; high accuracy (AUC up to 0.86); generalizable framework
Non-Invasive Decision Support for NSCLC Treatment Using PET/CT Radiomics	Mu et al., 2020 [19]	429 (training), 187 (validation) from Shanghai Pulmonary Hospital, SPH/Harbin Medical University, HBMU; multi-institutional cohorts (Shanghai, Hebei, Harbin)	Retrospective, multicentre	External validation (Harbin cohort)	Retrospective design; cross-site heterogeneity; small PD-L1 subgroup; potential selection bias	Multi-institutional; deep learning for EGFR mutation prediction; supports treatment selection (EGFR-TKI vs. ICI)
PET/CT-Based Cross-Modal DL Signature to Predict Occult Nodal Metastasis in Lung Cancer	Zhong et al., 2023 [20]	3265 total (1911 internal, 355 external, 999 prospective)	Retrospective + prospective multicentre	Internal, external, and prospective validation	Limited histologic subgroup analysis; computational complexity; inter-site variability	Very large sample; multi-cohort; prospective validation; excellent AUC (0.88–0.95); clinical translation readiness
Synthetic PET from CT Improves Diagnosis and Prognosis for Lung Cancer: Proof of Concept	Salehjahromi et al., 2024 [22]	1478 multicentre CT/PET pairs	Multicentre, retrospective deep learning (GAN-based)	External validation (MD Anderson + TCIA-Stanford)	Proof-of-concept only; retrospective; limited biological validation	Multicentre/multi-modalities; GAN-generated PET validated by radiologists and radio genomics; improved diagnosis and prognosis; potential to reduce PET cost and dose

Abbreviations—DL: deep learning, PET/CT: positron emission tomography/computed tomography, OSEM: ordered subset expectation maximisation, BSREM: block sequential regularised expectation maximisation, AUC: area under the curve, FD: full-dose, FDG: fluorodeoxyglucose, NSCLC: non-small-cell lung cancer, TCGA: the cancer genome atlas, TCIA: the cancer imaging archive, TIME: tumour immune microenvironment, IBSI: image biomarker standardisation initiative, FL: federated learning, RF: random forest, SVM: support vector machine, ANN: artificial neural network, CNN: convolutional neural network, PSMA-RADS: prostate-specific membrane antigen reporting data system, EGFR: epidermal growth factor receptor, EGFR-DLS: epidermal growth factor receptor Deep Learning signature, TKI: tyrosine kinase inhibitor, ICI: immune checkpoint inhibitor, HRCT: high resolution computed tomography, GAN: generative adversarial network.

## Data Availability

No new data were created or analysed in this study.
